# Enhanced effect of recombinant adenoviruses co‐expression of *ING4* and *OSM* on anti‐tumour activity of laryngeal cancer

**DOI:** 10.1111/jcmm.17192

**Published:** 2022-01-24

**Authors:** Fuwei Cheng, Shuangping Zhao, Jiachen Li, Yuyu Niu, Haiping Huang, Jicheng Yang, Shiyin Ma, Jisheng Liu, Peng Sun

**Affiliations:** ^1^ Department of Otolaryngology The First Affiliated Hospital of Soochow University Suzhou China; ^2^ Cell and Molecular Biology Institute College of Medicine Soochow University Suzhou China; ^3^ Department of Otolaryngology The First Affiliated Hospital of Bengbu Medical College Bengbu China

**Keywords:** gene therapy, growth family member 4, laryngeal cancer, Oncostatin M

## Abstract

The inhibitor of growth family member 4 (*ING4*) is one of the ING family genes, serves as a repressor of angiogenesis or tumour growth and suppresses loss of contact inhibition. Oncostatin M (OSM) is a multifunctional cytokine that belongs to the interleukin (IL)‐6 subfamily with several biological activities. However, the role of recombinant adenoviruses co‐expressing *ING4* and *OSM* (Ad‐ING4‐OSM) in anti‐tumour activity of laryngeal cancer has not yet been identified. Recombinant Ad‐ING4‐OSM was used to evaluate their combined effect on enhanced anti‐tumour activity in Hep‐2 cells of laryngeal cancer in vivo. Moreover, in vitro function assays of co‐expression of Ad‐ING4‐OSM were performed to explore impact of co‐expression of Ad‐ING4‐OSM on biological phenotype of laryngeal cancer cell line, that is Hep‐2 cells. In vitro, Ad‐ING4‐OSM significantly inhibited the growth, enhanced apoptosis, altered cell cycle with G1 and G2/M phase arrest, and upregulated the expression of P21, P27, P53 and downregulated survivin in laryngeal cancer Hep‐2 cells. Furthermore, in vivo functional experiments of co‐expressing of Ad‐ING4‐OSM demonstrated that solid tumours in the nude mouse model were significantly suppressed, and the co‐expressing Ad‐ING4‐OSM showed a significant upregulation expression of P21, P53, Bax and Caspase‐3 and a downregulation of Cox‐2, Bcl‐2 and CD34. This study for the first time demonstrated the clinical value and the role of co‐expressing Ad‐ING4‐OSM in biological function of laryngeal cancer. This work suggested that co‐expressing Ad‐ING4‐OSM might serve as a potential therapeutic target for laryngeal cancer patients.

## 1 INTRODUCTION

The inhibitor of growth family member 4 (*ING4*) is one of the ING family genes, a novel tumour suppressor gene family. *ING4* is located at chromosome 12p13.31, consists of eight exons and encodes a 29‐kDa protein expressed in multiple human tissues.^1^, ^2^, ^3^ ING4 was downregulated in many human cancer cells, such as glioblastoma^1^ and hepatocellular carcinoma.^4^ The allelic loss of *ING4* locus has been reported in breast cancer.^2^, ^5^ As a candidate tumour suppressor, it plays a critical role in repressing cell proliferation,^6^ tumour growth,^7^ loss of contact inhibition^2^ and angiogenesis.^8^ Oncostatin M (OSM) is a multifunctional cytokine that belongs to the interleukin (IL)‐6 subfamily. Human OSM (hOSM) was initially recognized by its activity to inhibit the proliferation of A375 melanoma cells and numerous other tumour cells.^9^ hOSM is a secreted glycoprotein of 28 kDa that was originally isolated from phorbol 12‐myristate 13‐acetate (PMA)‐stimulated human histiocytic lymphoma U937 cells. Furthermore, OSM is a unique cytokine that functions in various biological systems, such as inflammatory response, haematopoiesis, tissue remodelling and development.^10^ It also inhibits tumour cell growth and induces cell cycle alteration and apoptosis in different tumour types, such as melanomas,^11^, ^12^ glioblastomas,^13^ lung carcinomas,^14^ ovarian carcinomas^15^ and breast tumours.^16^, ^17^ In addition, human OSM induces differentiation of several tumour cell types.^13^, ^18^

Laryngeal cancer is the most common type of head and neck cancer in most countries; tobacco smoking and alcohol consumption are the major risk factors. The common treatments include surgery, chemotherapy, radiation and combinations of two or three of the above methods. The effects of ING4 and OSM emphasize their potential application as gene therapeutic agents. In this study, we explored the role of ING4 and OSM on enhanced anti‐tumour activity for human laryngeal cancer in vitro and in vivo and also elucidation of the underlying mechanism.

## 2 MATERIALS AND METHODS

### 2.1 Vectors, cell lines and mice

The Ad‐green fluorescent protein (Ad‐GFP), Ad‐ING4, Ad‐OSM and Ad‐ING4‐OSM replication‐incompetent Ad5E1‐ and E3‐deleted adenoviruses were constructed from the Cell and Molecular Biology Institute, College of Medicine, Soochow University (Suzhou, China). The Hep‐2 human laryngeal cancer cell line was supplied by the Cell Bank of Type Culture Collection of Chinese Academy of Sciences (Shanghai, China) in April 2019. Cells were grown in culture medium (DMEM containing 10% FBS and 100 U/ml of penicillin‐streptomycin antibiotics), identified by short tandem repeat (STR) profiling (Genetic Testing Biotechnology, Suzhou, Jiangsu, China) and confirmed to have no mycoplasma contamination. We tested the Hep‐2 human laryngeal cancer cell line in June 2019 at the last time. The Hep‐2 human laryngeal cancer cell line was cultured in RPMI1640 (Gibco) supplemented with 10% foetal bovine serum (FBS; Hyclone). The 3‐(4,5‐dimethylthiazol‐2‐yl)‐2,5‐diphenyltetrazolium bromide (MTT) kit was purchased from Sigma. Hoechst 33258 dye, cell cycle detection and Annexin V‐PE/7‐AAD apoptosis detection kits were purchased from KeyGene Biotech. The RT‐PCR primers, the antibodies against β‐actin, ING4, OSM, P21, P53, Bax, Bcl‐2, Survivin, Cox‐2 and CD34 were purchased from Abcam. Male athymic nude mice were purchased from Shanghai Experimental Animal Center and maintained in the Animal Facility at Soochow University according to the Animal Research Committee guidelines of Soochow University.

### 2.2 In vitro treatment

The experiment was designed with following five groups: (1) Phosphate‐buffered saline (PBS): treated with PBS as a cell control; (2) Ad‐GFP: treated with Ad‐GFP at the optimal multiplicity of infections as a blank adenovirus (Ad) control; (3) Ad‐ING4: treated with Ad‐ING4 at the optimal multiplicity of infections; (4) Ad‐OSM: treated with Ad‐OSM at the optimal multiplicity of infections; and (5) Ad‐ING4‐OSM: treated with Ad‐ING4‐OSM at the optimal multiplicity of infections. For each group, the cells were cultured in RPMI1640 supplemented with 10% FBS incubated overnight at 37°C in humidified 5% CO_2_ atmosphere. After treatment with gene recombinant adenoviruses, 2% FBS was used instead.

### 2.3 Adenoviral infection efficiency

In order to assess the optimal multiplicity of infections for a maximal transgene expression and no cytotoxicity, Hep‐2 cells were infected with Ad‐GFP, Ad‐ING4, Ad‐OSM and Ad‐ING4‐OSM, at 10, 25, 50, 75, 100 and 200 multiplicity of infections, respectively, cultured after 48 h and examined by fluorescence microscopy.

### 2.4 Ad‐ING4‐OSM transgene expression

The Ad‐directed ING4 and OSM transgene expression in Hep‐2 cells was analysed by RT‐PCR and Western blot. For the RT‐PCR, the total cellular RNA was extracted using TRIzol, and the first‐strand cDNA was reverse transcribed with RNA as a template and Oligo d(T)_18_ as a primer. The PCR amplification was carried out using cDNA as the template and primers specific for *ING4* and *OSM*, respectively. For the Western blot, proteins were isolated from infected and uninfected Hep‐2 cells (1–2 × 10^6^), resolved by 12% sulphate‐polyacrylamide gel electrophoresis (SDS‐PAGE) and transferred onto a polyvinylidene difluoride (PVDF) membrane. After blocking with 5% (w/v) nonfat dry milk in tris‐buffered saline containing 0.05% Tween 20 (TBST), the membrane was probed with primary antibody polyclonal mouse anti‐ING4 (1:1000) or anti‐OSM (1:1000) in blocking solution for 2 h, followed by incubation with horseradish peroxidase (HRP)‐conjugated secondary antibody (rabbit anti‐mouse) for another 2 h. The membrane was developed using a SuperEnhanced chemiluminescence detection kit. The immunoreactive bands were visualized after exposure of the membranes to Kodak X‐ray film.

### 2.5 MTT (3‐(4,5‐dimethylthiazol‐2‐yl)‐2,5‐diphenyltetrazolium bromide) assay

The cytotoxic activity of each group of Hep‐2 cells was determined by MTT assay. Briefly, the Hep‐2 cells were dispensed in a 96‐well culture plate at a density of 0.5 × 10^4^ cells/well and incubated at 37°C in a humidified 5% CO_2_ atmosphere. After incubation for overnight, the cells were treated for 0–4 days. After treatments with different periods, the cells were incubated with 10 μl MTT (5 mg/ml) at 37°C for 4 h. The formazan crystals in the cells were solubilized with 10% SDS‐HCl (100 μl/well). The plate was read at 570 nm using a Microplate Reader Model 550 (Bio‐Rad). The cell inhibition rate was calculated as (A control group−A treatment group)/A control group × 100%.

### 2.6 Hochest 33258 fluorescence staining

The Hep‐2 cells from exponentially growing cultures were seeded in 24‐well culture plates for 24 h. After treatments, for each group, the cells were washed with ice‐cold PBS and fixed in a solution of methanol‐acetic acid (3:1, v/v) at 4°C for 15 min. The apoptotic Hep‐2 cells were identified by staining with Hoechst 33258 (5 μg/ml in PBS) for 15 min at room temperature in dark. The nuclei structure of the cells was examined under an Olympus fluorescence microscope with an excitation wavelength of 340 nm and an emission wavelength of 460 nm.

### 2.7 Flow cytometry of cell cycle by PI (propidium iodide)

After the Hep‐2 cells were treated, respectively, for 48 h, each group of cells (1 × 10^5^) were harvested, washed in cold PBS, fixed in 70% cold alcohol for >24 h at 4°C, washed in cold PBS and stained with PI solution containing 0.1 mg/ml RNase A at 4°C in dark for 30 min. The DNA content and cell cycle were analysed by flow cytometry.

### 2.8 Flow cytometry of cell apoptosis by Annexin V‐PE/7‐AAD

Apoptosis was assessed using Annexin V‐PE/7‐AAD double staining following manufacturer's instructions. For each group, the Hep‐2 cells were cultured 24 h and treated, respectively. Subsequently, 1 × 10^5^ cells were harvested, washed in cold PBS for twice, incubated for 15 min at room temperature in the presence of 5 μl Annexin V‐PE and 5 μl 7‐AAD in 100 μl of 1× binding buffer in dark. After incubation, 400 μl of 1 × binding buffer was added, and the apoptotic cells were analysed by flow cytometry.

### 2.9 Real‐time reverse transcription (RT)‐PCR

To further determine the expression levels of P21, P27, P53 and Survivin in Hep‐2 cells for each group, total RNA was prepared for the two‐step real‐time RT‐PCR analysis based on SYBR Green I detection. The RT‐PCR assay was performed using the MJ Research OpticonTM2 system (MJ Research). Briefly, total RNA was isolated using TRIzol reagent (Invitrogen). The first‐strand cDNA synthesis was performed as described above in larger volumes, such that each sample could be tested in different subsequent PCR reactions. The PCR reaction was performed using the following program: 95°C for 5 min, then 72℃ for 5 min, followed by 50 cycles of 95°C for 15 s, 58°C for 20 s and 72°C for 30 s. The cDNA quantities were normalized to that of the internal control gene *Gapdh* measured in the same samples. The relative gene expression of the target gene was calculated using the 2^−△△CT^ method with pooled cDNA from all samples as a reference.^19^ The authenticity of the PCR products was verified by melting curve analysis and agarose gel electrophoresis. Each sample was analysed in duplicate in independent reactions, and experiments were repeated at least three times.

### 2.10 Animal experiments

Male athymic nude mice were subcutaneously inoculated on the armpits of the right anterior limbs with 1–2 × 10^6^ Hep‐2 cells and monitored daily for tumour growth. The tumour volume was measured with a calliper and calculated by the formula below: tumour size = *ab* ^2^/2, where *a* is the larger and *b* is the smaller of the two dimensions. When the tumours grew to a mean tumour volume of 0.1–0.2 cm^3^, the Hep‐2 cells xenografted tumour‐bearing mice were intratumorally injected with PBS (PBS control) or 1 × 10^8^ plaque‐forming units (pfu) of Ad‐GFP, Ad‐ING4, Ad‐OSM and Ad‐ING4‐OSM every other day for five times, respectively. Tumour progression and regression were monitored, and tumour volume was measured every alternate day. In addition, the tumour‐bearing mice (5 in each group) were sacrificed 4 days after final treatment, and then, the xenografted tumours were removed, weighed, fixed by 10% neutral formalin and embedded in paraffin for haematoxylin‐eosin (HE) staining and immunohistochemistry.

### 2.11 Immunohistochemistry

The expression of P21, P53, Survivin, Bax, Bcl‐2, CD34, Cox‐2 and Caspase‐3 in Hep‐2 cells xenografted tumours was tested, respectively, by immunohistochemistry using UltraSensitive^™^ SP kit according to the manufacturer's protocols. The presence of buffy or brown diaminobenzidine precipitates indicated positive reactivity. Then, the integral optical density (IOD) of immunohistochemistry was calculated by Image‐Pro Plus 6.0 software. Each value represents IOD at a power view (200×) by microscopy. The mean value represents the average number derived from five high‐power fields in each group.

### 2.12 Statistical analysis

All data were presented as the mean ± SD. The significance of differences between groups was evaluated by one‐way and two‐way repeated measure analysis of variance (ANOVA) and multiple comparisons using SPSS 17.0 software, and *p* < 0.05 (95% confidence interval) indicated a statistical significance.

In order to analyse whether ING4 and OSM have a synergistic effect on anti‐tumour activity, we used the method of Jin et al. as a standard by calculation of Q [20]. Q = E(A+B)/(EA + EB−EA × EB). EA or EB was the single effect, E(A + B) was the real combined effect, while (EA + EB−EA × EB) was the expected combined effect. If Q = 1 ± 0.15, then the two factors A and B were considered having summation action; if Q > 1.15, then A and B were considered to have synergistic action; and if Q < 0.85, then A and B were considered as an antagonistic action.

## 3 RESULTS

### 3.1 Adenoviral infection efficiency

**FIGURE 1:**
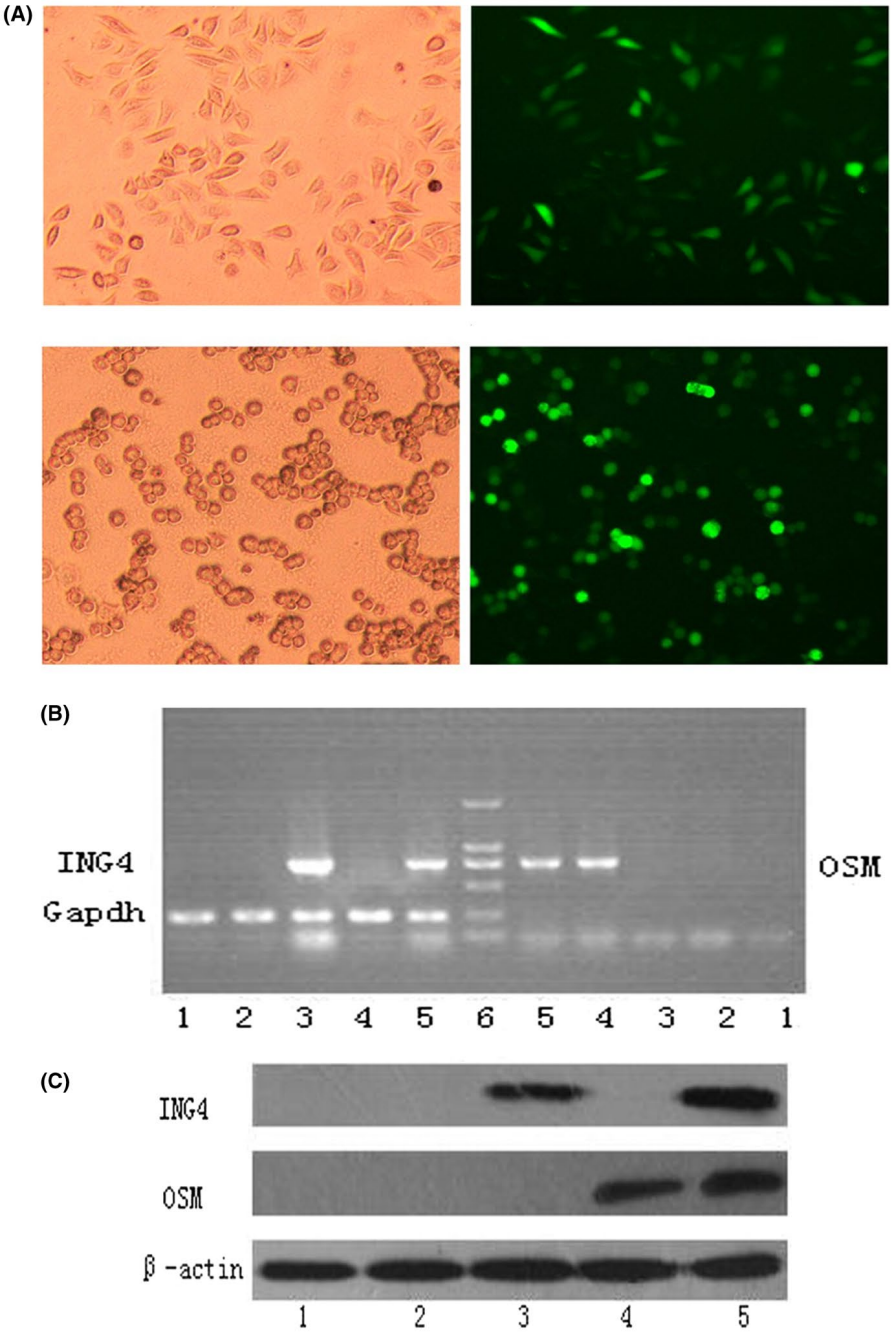
Light microscopy and fluorescence microscopy photograph of Hep‐2 cells. *ING4* and *OSM* transgene expression in Hep‐2 cells. (A) Hep‐2 cells (fusiform and normal) were infected with Ad‐GFP and showed almost no apoptosis. (B) Apoptotic Hep‐2 cells (round and abnormal) infected with Ad‐ING4, Ad‐ OSM, Ad‐ING4‐OSM or excess Ad‐GFP. (C). RT‐PCR analysis (D). Western blot analysis. Lane 1: PBS group; lane 2: Ad‐GFP group; lane 3: Ad‐ING4 group; lane 4: Ad‐OSM group; lane 5: Ad‐ING4‐OSM group; lane 6: DNA marker (2 kbp)

The human laryngeal cancer Hep‐2 cells were infected with Ad, Ad‐ING4, Ad‐OSM and Ad‐ING4‐OSM at 10, 25, 50, 75, 100 and 200, respectively, for 48 h, and then examined by fluorescence microscopy. More than 90% of GFP expression was found in the Ad‐infected Hep‐2 cells at optimal multiplicity of infections ≥100, whereas the GFP expression was not detected in the uninfected Hep‐2 cells. At 100 MOI, Ad showed cytotoxicity to Hep‐2 cells seldom, while at 200 MOI, Ad makes the cells apoptotic as shown in Figure 1A.

### 3.2 Ad‐ING4‐OSM transgene expression

The human laryngeal cancer Hep‐2 cells were infected with Ad, Ad‐ING4, Ad‐OSM and Ad‐ING4‐OSM at 100 MOI and cultured for 48 h. The RT‐PCR analysis of the transcriptional expression of transgene ING4 and OSM is shown in Figure 1B. As expected, transgene ING4 was detected in Ad‐ING4 and Ad‐ING4‐OSM groups, and transgene OSM was detected in Ad‐OSM and Ad‐ING4‐OSM groups. Western blot analysis (Figure 1C) showed that a significant amount of ING4 expression was detected in Ad‐ING4 and Ad‐ING4‐OSM groups, and a significant expression of OSM was found in the Ad‐OSM and Ad‐ING4‐OSM groups, but not in the other groups. The RT‐PCR and Western blot indicated that transgene ING4 and OSM were successfully inserted into Hep‐2 cells and expressed.

### 3.3 Ad‐ING4, Ad‐OSM and Ad‐ING4‐OSM had inhibitory effect of Hep‐2 cells in vitro

**FIGURE 2:**
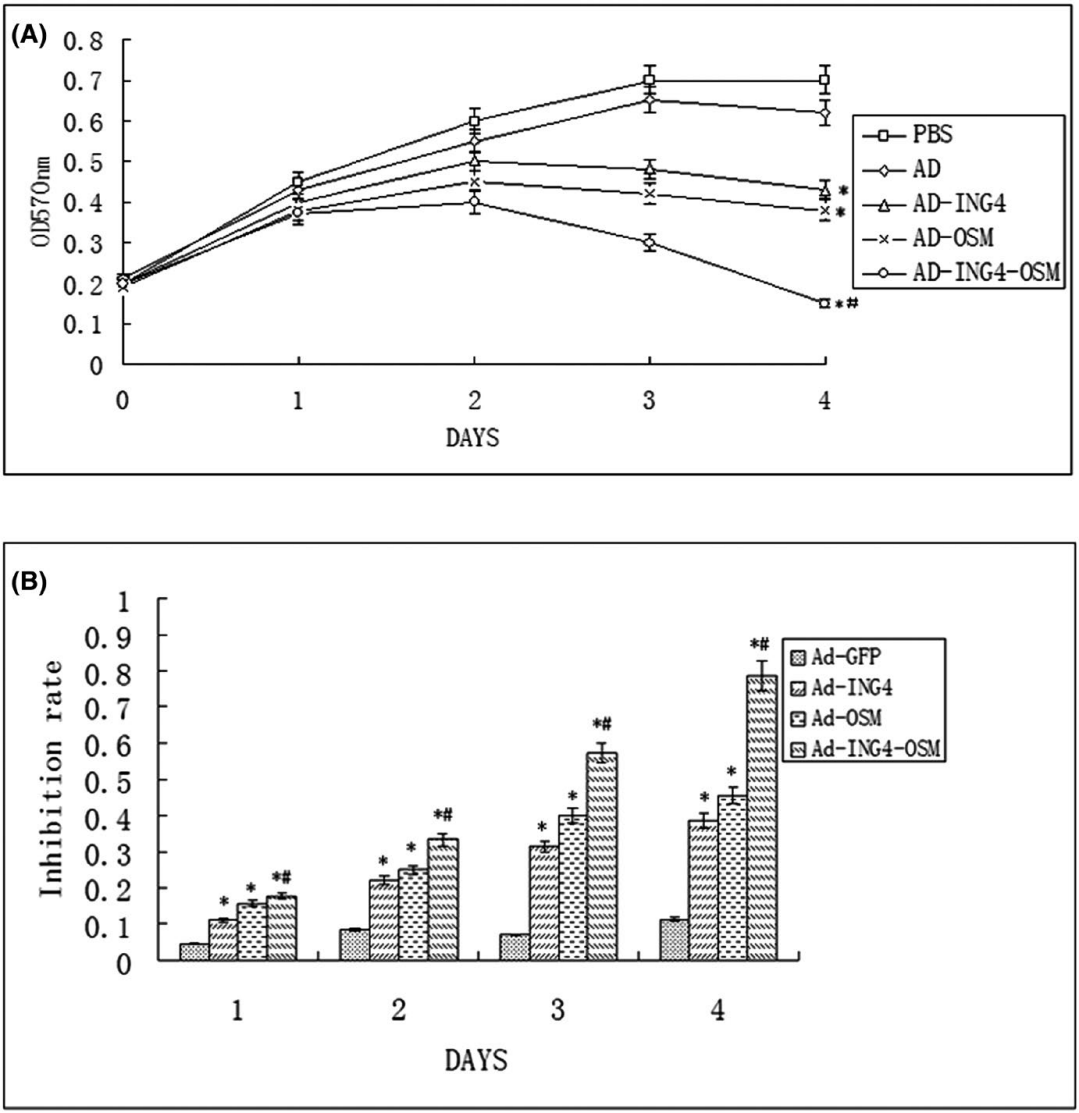
(A and B) Inhibitory effect of Ad‐ING4‐OSM on Hep‐2 cells. Compared to PBS and Ad‐GFP groups, *p < 0.05; compared to the Ad‐ING4 and Ad‐OSM groups, ^#^ *p* < 0.05

For each group, the Hep‐2 cells were treated at the indicated periods (0–4 days), respectively. The cell viability was evaluated at days 0, 1, 2, 3 and 4 after MTT assay, respectively (Figure 2A,B). On the day 4, Ad had little, while Ad‐ING4, Ad‐OSM and Ad‐ING4‐OSM had obvious inhibitory action, and the inhibition rate was 39%, 46% and 79%, respectively. There was no significant difference in inhibitory action between the Ad and PBS groups (*p* > 0.05), while the differences were significant between Ad‐ING4, Ad‐OSM and Ad‐ING4‐OSM groups compared to the PBS and Ad groups (*p* < 0.05). Moreover, such a significant difference existed for Ad‐ING4‐OSM group compared to Ad‐ING4 and Ad‐OSM groups (*p* < 0.05). Simultaneously, Q = 1.18 confirmed a synergistic action in the Ad‐ING4‐OSM group.

### 3.4 Ad‐ING4‐OSM arrested Hep‐2 cells in both G2/M and G0/G1 phase while decrease the proportion of the S phase by PI

**FIGURE 3:**
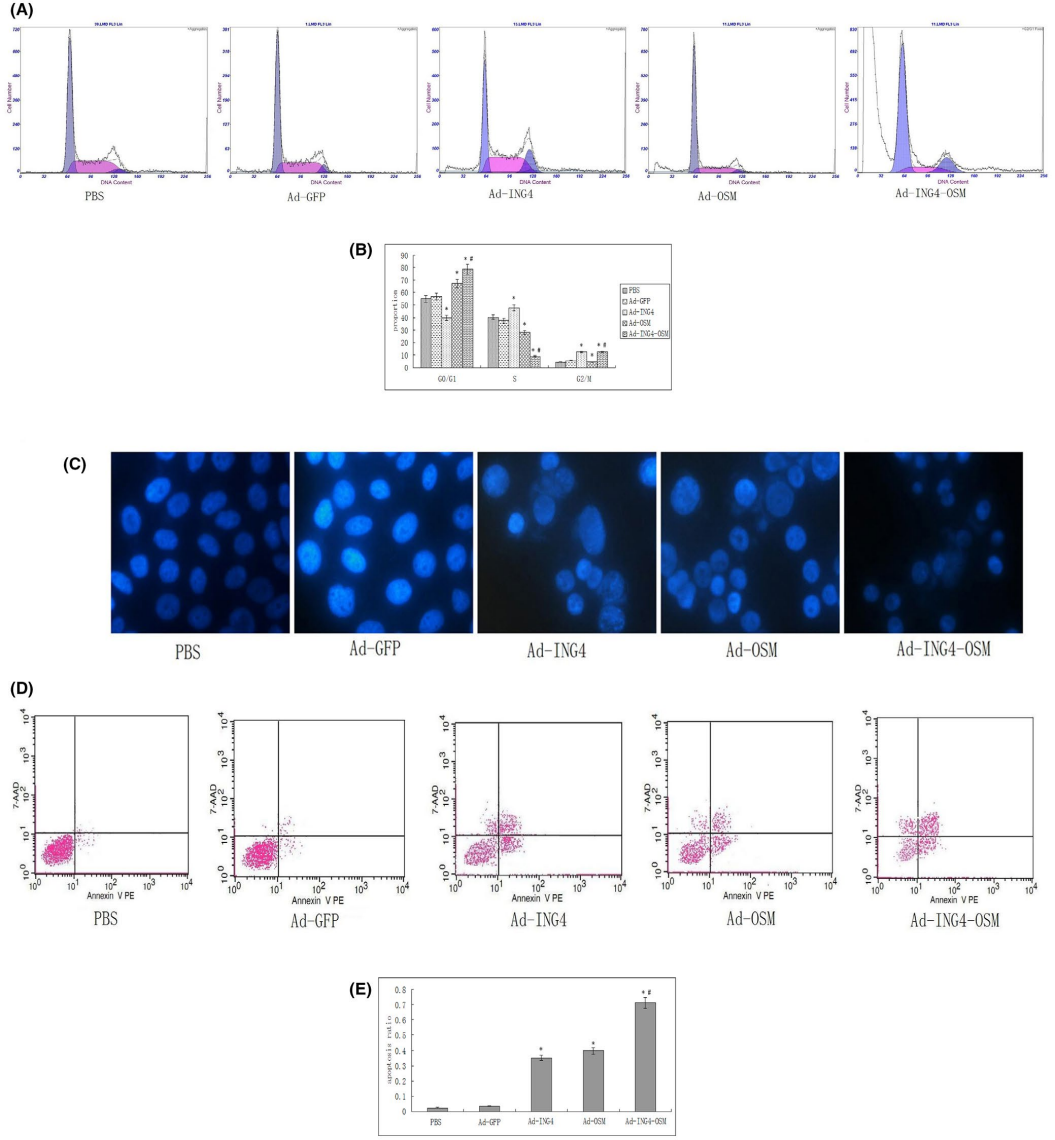
(A and B) Cell cycle phase analysis (compared to the PBS and Ad‐GFP groups, *p < 0.05; compared to the Ad‐ING4 and Ad‐OSM groups, ^#^ *p* < 0.05). (C) Hoechst 33258 fluorescent staining for Hep‐2 cells. (D and E) Apoptosis analysis of Hep‐2 cells (compared to PBS and Ad‐GFP groups, *p < 0.05; compared to the Ad‐ING4 and Ad‐OSM groups, ^#^ *p* < 0.05)

To further explore the mechanisms of Hep‐2 cell growth inhibition, we analysed the cell cycle distribution, as assessed by PI staining shown in Figure 3A,B, the Ad‐ING4 arrested Hep‐2 cells in the G2/M phase, and the proportion of the G2/M phase was 12.3% ± 2.2% higher than the PBS and Ad‐GFP groups (4.6% ± 1.0% and 5.5% ± 1.4%, respectively; *p* < 0.05). Ad‐OSM arrested Hep‐2 cells in G0/G1 phase, and the proportion of the G0/G1 phase was 67.1% ± 2.5%, which was higher than those of PBS and Ad‐GFP (55.1% ± 1.7% and 56.8% ± 1.9%, respectively; *p* < 0.05). Ad‐ING4‐OSM arrested Hep‐2 cells in both G2/M and G0/G1 phases; and the proportion of was 12.3% ± 1.0%) for the G2/M phase and 78.6% ± 2.3% for the G0/G1 phase. After treatment with Ad‐ING4‐OSM, the proportion of G2/M and G0/G1 phase cells was increased, while the proportion of the S phase cells was markedly decreased compared to the control group, suggesting that Hep‐2 cells were less proliferative.

### 3.5 Flow cytometry of cell apoptosis by Hoechst 33258 fluorescence staining and Annexin V‐PE/7‐AAD

The Hep‐2 cells in each group were stained with fluorescent dye Hoechst 33528 and visualized under a fluorescence microscope. Hoechst nuclear staining binds to the AT‐rich regions of double‐stranded DNA and exhibits enhanced fluorescence. Hoechst dye permeabilizes through the intact membranes of Hep‐2 cells and stain the DNA. Figure 3C shows the results of Hoechst staining. PBS and Ad exhibited round normal nuclei. Conversely, Ad‐ING4, Ad‐OSM and Ad‐ING4‐OSM showed signs of apoptosis, including highly condensed and fragmented nuclei. Moreover, the cells treated with Ad‐ING4‐OSM also exhibited more apoptotic cells than those in the Ad‐ING4 and Ad‐OSM groups.

To explore the apoptosis of Hep‐2 cells, we used Annexin V‐PE/7‐AAD double staining by flow cytometry. As shown in Figure 3D,E, the apoptotic ratio was 2.3% and 3.5% in the PBS and Ad‐GFP groups, respectively, with no significant difference (*p* > 0.05). However, the apoptotic rates in Hep‐2 cells were 35.2%, 39.7% and 71.2% in the Ad‐ING4, Ad‐OSM and Ad‐ING4‐OSM groups, respectively. Moreover, the apoptotic rates were significant different between Ad‐ING4, Ad‐OSM and Ad‐ING4‐OSM groups compared to those in the PBS and Ad group (*p* < 0.05). When compared to the Ad‐ING4 and Ad‐OSM groups, the Ad‐ING4‐OSM was found to significantly induce Hep‐2 cell apoptosis (*p* < 0.05) with a synergistic effect (Q = 1.17).

### 3.6 Ad‐ING4, Ad‐OSM and Ad‐ING4‐OSM group result in upexpression of P21, P27 and P53 and downexpression of survivin by real‐time RT‐PCR

**FIGURE 4:**
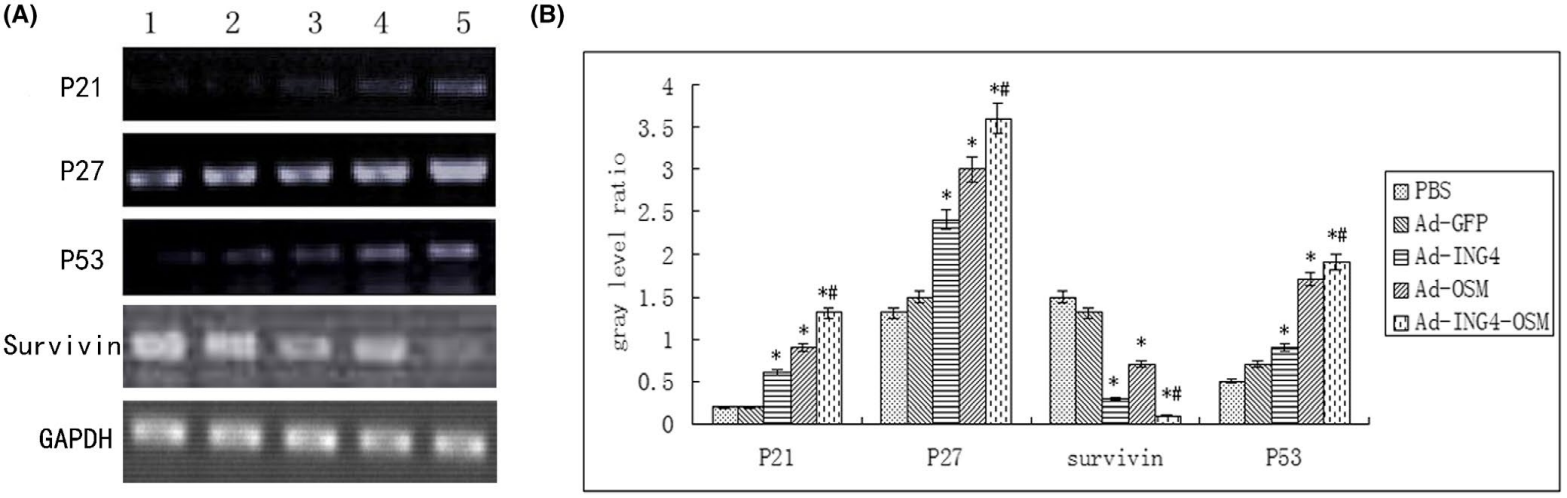
Expression levels of P21, P27, P53 and Survivin analysed by RT‐PCR. Lane 1: PBS groups; lane 2: Ad‐GFP groups; lane 3: Ad‐ING4 group; lane 4: Ad‐OSM group; lane 5: Ad‐ING4‐OSM group

The real‐time RT‐PCR analysed the expression levels of P21, P27, P53 and survivin (Figure 4A,B). Compared to the PBS group, the Ad group had little effect on gene expression, while the Ad‐ING4 and Ad‐OSM groups significantly resulted in upexpression of P21, P27 and P53 and downexpression of survivin. Compared to the Ad‐ING4 and Ad‐OSM group, the Ad‐ING4‐OSM group had a remark increase in gene expression.

### 3.7 Ad‐ING4‐OSM suppressed Hep‐2 tumour growth in vivo

**FIGURE 5:**
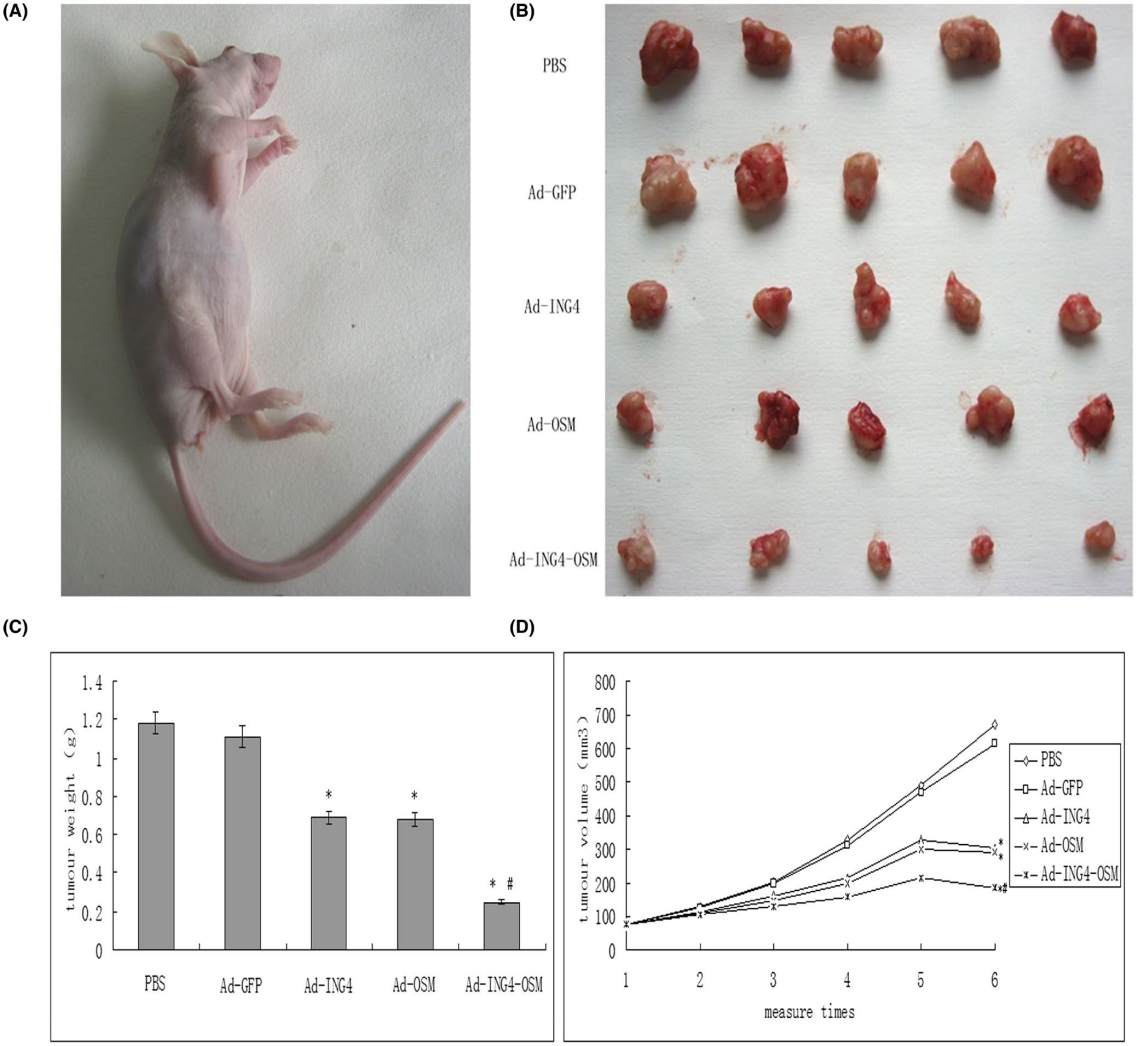
Tumours in male athymic nude mice and the change in tumour weight and volume (compared to PBS and Ad‐GFP groups, *p < 0.05; compared to Ad‐ING4 and Ad‐OSM groups, ^#^ *p* < 0.05)

Male athymic nude mice were subcutaneously inoculated on the armpits of right anterior limbs with Hep‐2 cells (2 × 10^6^ cells/mouse). At 2 weeks after inoculation, all animals survived and had tumour growth, as shown in Figure 5A. After treatment with PBS, Ad, Ad‐ING4, Ad‐OSM and Ad‐ING4‐OSM, respectively, the tumours were excised (Figure 5B), the change in tumour weight and volume is shown in Figure 5C,D, and Ad‐ING4‐OSM remarkably suppressed Hep‐2 tumour growth in vivo compared to Ad‐ING4 and Ad‐OSM groups, while Ad exerted a slight suppression effect compared to PBS.

### 3.8 Ad‐ING4, Ad‐OSM and Ad‐ING4‐OSM upregulate P21, P53 and Caspase‐3 expression and downregulate Cox‐2, Bcl‐2 and CD34 expression by immunohistochemistry in Hep‐2 cell‐xenografted tumours

**FIGURE 6:**
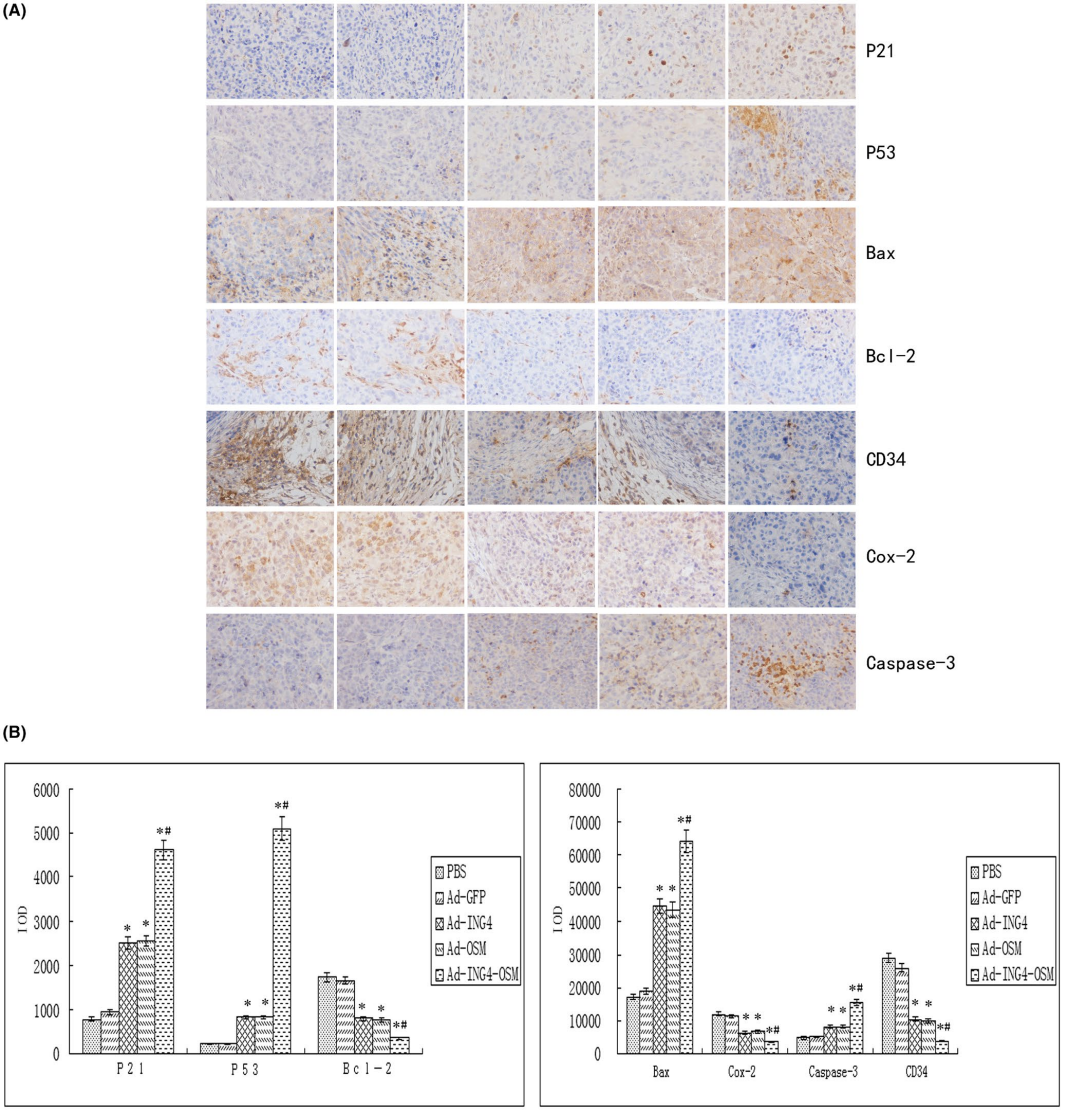
Expression of P21, P53, Bax, Bcl‐2, CD34, Cox‐2 and Caspase‐3, respectively, in Hep‐2 cells xenografted tumours tested by immunohistochemistry analysis

The expression of P21, P53, Bax, Bcl‐2, CD34, Cox‐2 and Caspase‐3 in Hep‐2 cell‐xenografted tumours was evaluated by immunohistochemistry mainly represented as brownish yellow or brownish granules as shown in Figure 6A. Compared to the Ad and PBS groups, the expression of P21, P53, Bax and Caspase‐3 in the Ad‐ING4, Ad‐OSM and Ad‐ING4‐OSM groups was stronger while the expression of Cox‐2, Bcl‐2 and CD34 was weaker, indicating that ING4 and/or OSM might upregulate P21, P53, Bax and Caspase‐3 expression and downregulate Cox‐2, Bcl‐2 and CD34 expression, respectively, in Hep‐2 cell‐xenografted tumours. The IOD (Figure 6B) in the Ad‐ING4, Ad‐OSM, and Ad‐ING4‐OSM groups was significantly more or less than that in the Ad and PBS groups, respectively (*p* < 0.05). Furthermore, Ad‐ING4‐OSM had a synergistic effect.

## 4 DISCUSSION

Since gene therapy represents a rational and potentially successful treatment for cancers, recombinant Ad vectors provide a highly versatile system for mammalian gene transfer and are widely used in vaccine development and a variety of gene therapy applications, especially for gene‐based therapy of cancer. Currently, up to 600 gene therapy clinical protocols have been reported, of which 28% used Ad to deliver the therapeutic or marker genes. The biology of recombinant Ad has been studied extensively and is well‐understood. Compared to other vectors, Ad has several advantages,^20^ including (1) convenient and simple methods of vector construction; (2) efficient transduction of proliferating and quiescent cells; (3) efficient production to high yields in well‐defined cell systems; and (4) high stability, allowing purification and long‐term storage. In the current study, a recombinant replication‐deficient Ad that expresses a mutant of *Aquorea victoria* green fluorescent protein (GFP) has been used to transfer ING4 and OSM.

Inhibitor of growth family member 4 is a candidate tumour suppressor gene that exerts the tumour‐suppressive effect via multiple pathways in various tumours.^21^ A recent study showed that Ad‐ING4‐P53 and cisplatin gene therapy in hypopharyngeal cancer is associated with apoptosis induction.^21^, ^22^ ING4 regulates the p65 subunit of nuclear factor (NF)‐кB via interaction with Sirtuin1 (SIRT1) and alleviates lipopolysaccharide‐induced inflammation.^23^ ING4 inhibits tumour proliferation and angiogenesis in human glioma.^24^ Moreover, ING4 downregulates IL‐6, IL‐8, MMP‐2 and MMP‐9 expression in the human lung adenocarcinoma cell line A549^25^ and inhibits tumour cell growth and induces cell cycle alteration and apoptosis in various tumour types,^6^, ^21^, ^25^, ^26^, ^27^, ^28^, ^29^ such as hepatocellular, melanoma, colorectal, glioblastoma, breast and lung cancer. In addition, it enhances chemosensitivity in HepG2 hepatocarcinoma cells.^26^ A recent study reported that ING4 exerts a marked inhibitory effect on tumour cell spread, migration and invasion.^7^, ^30^

Oncostatin M plays a critical role in various biological systems, such as inflammatory response, haematopoiesis, tissue remodelling and development.^10^ OSM receptor beta (OSMR‐β) deficiency attenuates atherogenesis by inhibiting the JAK2/STAT3 signal pathway in macrophages,^31^ and the type II OSMR consists of gp130 and the OSM‐specific receptor subunit.^32^, ^33^ OSM activates intracellular signalling cascades through OSMR containing gp130. The interactions between OSM and its receptor complex (gp130‐OSMR‐β) trigger the activation of two main signalling pathways: the JAK‐signal transducer and activator of transcription (STAT) (with STAT1, STAT3 and STAT5) and the STAT3‐SMAD3.^10^, ^34^ Furthermore, OSM induces STAT3 and ERK signalling and promotes the proliferation and migration of malignant keratinocyte PDVC57 cells.^35^ These phenomena suggested that growth inhibition in these cells by cytokines could be attributed to the suppressed activity of cyclin‐dependent kinase (CDK) by p27kip1. Recent studies implicated OSM in macrophage M2 polarization, which might promote tumour progression.^36^, ^37^, ^38^ OSM reportedly increases the level of metastasis‐related proteins, including matrix metalloproteinase‐1 (MMP‐1) in osteogenic differentiation of mouse MC3T3 osteoblasts,^39^ MMP‐2 in human trophoblast cell line^40^ and cathepsin L in osteosarcoma cells.^41^ Moreover, OSM is cytostatic for high‐grade chondrosarcomas, independent of p53 and presumably through the JAK3/STAT1 pathway.^42^

Both ING4 and OSM exert a negative effect on cancer cells rather than normal cells. Han et al.^43^ have reported that inhibition of Adenovirus‐ING4‐OSM therapy on nasopharyngeal carcinoma proliferation in vitro and in vivo. In order to prove whether ING4 and OSM have a significant influence on the growth and apoptosis of human laryngeal cancer Hep‐2 cells, we used recombinant Ad vectors for gene transfer. First, we examined the successful expression of ING4 and OSM in Hep‐2 cells. Then, in vitro MTT assay and flow cytometric analysis of cell apoptosis by Annexin V‐PE/7‐AAD indicated that Hep‐2 cells with Ad‐*ING4* or Ad‐*OSM* gene alone exhibited growth inhibition and enhanced apoptosis, indicating that Ad‐ING4‐OSM effectuated synergistically. The mechanism underlying ING4‐ and OSM‐mediated negative regulation of tumour growth remains largely unknown. Thus, we analysed the molecular basis of the inhibitory effect of Ad‐ING4‐OSM on Hep‐2 cells. The cell cycle phase distribution by PI staining revealed that Ad‐ING4 arrested Hep‐2 cells in the G2/M phase, Ad‐OSM arrested Hep‐2 cells in G0/G1 phase and Ad‐ING4‐OSM arrested Hep‐2 cells in both G2/M and G0/G1 phases. Compared to Ad‐ING4 and Ad‐OSM groups, both RT‐PCR and immunohistochemistry analysis showed that Ad‐ING4‐OSM had an additive effect on the upregulation of P21, P27, P53, Bax and Caspase‐3 and on the downregulation of Survivin, Cox‐2, Bcl‐2 and CD34 in Hep‐2 cells. Together, these results indicated that Ad‐ING4‐OSM is a potential treatment strategy for Hep‐2 cells.

Nevertheless, there are several limitations in our study. First, the in vitro assays were only performed in Hep‐2 cells, which is the only laryngeal cancer cell line available in China for now. Second, the clinical value of recombinant adenoviruses co‐expression of *ING4* and *OSM* was not investigated in a large patient cohort. Thus, large multicentre studies including large cohorts of laryngeal cancer patients are warranted. Finally, the underlying mechanism of recombinant adenoviruses co‐expression of *ING4* and *OSM* in laryngeal cancer has not been fully clarified in this study. Therefore, we will validate these findings and expand our investigation by including additional laryngeal cancer cell lines in future.

## 5 CONCLUSION

Overall, our study suggests that recombinant adenoviruses co‐expression of *ING4* and *OSM* that in vitro, Ad‐ING4‐OSM significantly inhibited the growth, enhanced apoptosis, altered cell cycle with G1 and G2/M phase arrest and downregulated the expression of P21, P27, P53 and survivin in laryngeal cancer Hep‐2 cells. Furthermore, in vivo functional experiments of co‐expressing of Ad‐ING4‐OSM demonstrated that solid tumours in the nude mouse model were significantly suppressed, and the co‐expressing Ad‐ING4‐OSM showed a significant upregulation expression of P21, P53, Bax and Caspase‐3 and a downregulation of Cox‐2, Bcl‐2 and CD34. Therefore, co‐expression of Ad‐ING4‐OSM might serve as a novel therapeutic strategy for patients with laryngeal cancer.

## CONFLICT OF INTEREST

We have no competing interests.

## AUTHOR CONTRIBUTIONS

**fuwei cheng:** Writing – original draft (equal). **shuangping zhao:** Writing – original draft (equal). **jiachen li:** Methodology (equal). **yuyu niu:** Data curation (equal). **haiping huang:** Methodology (equal). **Jicheng Yang:** Formal analysis (equal). **shiyin ma:** Project administration (equal). **jisheng liu:** Funding acquisition (equal). **peng sun:** Funding acquisition (equal); Methodology (lead).

## References

[1] Shao B , Liu E . Experssion of ING4 is negatively correlated with cellular proliferation and microvessel density in human glioma. Oncol Lett. 2017;14:3663‐3668. 28927128 10.3892/ol.2017.6618 PMC5587956

[2] Kim S , Chin K , Gray JW , Bishop JM . A screen for genes that suppress loss of contact inhibition: identification of ING4 as a candidate tumor suppressor gene in human cancer. Proc Natl Acad Sci USA. 2004;101:16251‐16256. 15528276 10.1073/pnas.0407158101 PMC528940

[3] He GH , Helbing CC , Wagner MJ , Sensen CW , Riabowol K . Phylogenetic analysis of the ING family of PHD finger proteins. Mol Biol Evol. 2005;22:104‐116. 15356280 10.1093/molbev/msh256

[4] Qian F , Hu Q , Tian Y , et al. ING4 suppresses hepatocellular carcinoma via a NF‐kappaB/miR‐155/FOXO3a signaling axis. Int J Biol Sci. 2019;15:369‐385. 30745827 10.7150/ijbs.28422 PMC6367549

[5] Shatnawi A , Ayoub NM , Alkhalifa AE . ING4 expression landscape and association with clinicopathologic characteristics in breast cancer. Clin Breast Cancer. 2021;21(4):e319‐e331. 33334698 10.1016/j.clbc.2020.11.011

[6] Shen JC , Unoki M , Ythier D , et al. Inhibitor of growth 4 suppresses cell spreading and cell migration by interacting with a novel binding partner, liprin alpha1. Cancer Res. 2007;67:2552‐2558. 17363573 10.1158/0008-5472.CAN-06-3870 PMC2569966

[7] Qu H , Yin H , Yan S , Tao M , Xie Y , Chen W . Inhibitor of growth 4 suppresses colorectal cancer growth and invasion by inducing G1 arrest, inhibiting tumor angiogenesis and reversing epithelial‐mesenchymal transition. Oncol Rep. 2016;35:2927‐2935. 26936485 10.3892/or.2016.4626

[8] Chen Y , Huang Y , Hou P , et al. ING4 suppresses tumor angiogenesis and functions as a prognostic marker in human colorectal cancer. Oncotarget. 2016;7:79017‐79031. 27806345 10.18632/oncotarget.12984 PMC5346695

[9] Zarling JM , Shoyab M , Marquardt H , Hanson MB , Lioubin MN , Todaro GJ . Oncostatin M: a growth regulator produced by differentiated histiocytic lymphoma cells. Proc Natl Acad Sci USA. 1986;83(24):9739‐9743. 3540948 10.1073/pnas.83.24.9739 PMC387216

[10] Masjedi A , Hajizadeh F , Dargani FB , et al. Oncostatin M: a mysterious cytokine in cancers. Int Immunopharmacol. 2021;90:107158. 33187910 10.1016/j.intimp.2020.107158

[11] Malik N , Kallestad JC , Gunderson NL , et al. Molecular cloning, sequence analysis, and functional expression of a novel growth regulator, oncostatin M. Mol Cell Biol. 1989;9:2847‐2853. 2779549 10.1128/mcb.9.7.2847-2853.1989 PMC362750

[12] Brown TJ , Lioubin MN , Marquardt HA . Purification and characterization of cytostatic lymphokines produced by activated human T lymphocytes. Synergistic antiproli‐ferative activity of transforming growth factor h1, interferon‐, and oncostatin M for human melanoma cells. J Immunol. 1987;139:2977‐2983. 3117884

[13] Guo Q , Guan G‐F , Cao J‐Y , et al. Overexpression of oncostatin M receptor regulates local immune response in glioblastoma. J Cell Physiol. 2019;234:15496‐15509. 10.1002/jcp.28197 30693511

[14] Pan C‐M , Wang M‐L , Chiou S‐H , Chen H‐Y , Wu C‐W . Oncostatin M suppresses metastasis of lung adenocarcinoma by inhibiting SLUG expression through coordination of STATs and PIASs signalings. Oncotarget. 2016;7(37):60395‐60406. 27486982 10.18632/oncotarget.10939 PMC5312391

[15] Ohata Y , Harada T , Fujii A , Yoshida S , Iwabe T , Terakawa N . Menstrual cycle‐specific inhibition of endometrial stromal cell proliferation by oncostatin M. Mol Hum Reprod. 2001;7:665‐670. 11420390 10.1093/molehr/7.7.665

[16] Doherty MR , Parvani JG , Tamagno I , et al. The opposing effects of interferon‐beta and oncostatin‐M as regulators of cancer stem cell plasticity in triple‐negative breast cancer. Breast Cancer Res. 2019;21:54. 31036052 10.1186/s13058-019-1136-x PMC6489282

[17] Liu J , Hadjokas N , Mosley B , Estrov Z , Spence MJ , Vestal RE . Oncostatin M‐specific receptor expression and function in regulating cell proliferation of normal and malignant mammary epithelial cells. Cytokine. 1998;10:295‐302. 9617575 10.1006/cyto.1997.0283

[18] Douglas AM , Grant SL , Gross GA , Clouston DR , Sutherland RL , Begley CG . Oncostatin M induces the differentiation of breast cancer cells. Int J Cancer. 1998;75:64‐73. 9426692 10.1002/(sici)1097-0215(19980105)75:1<64::aid-ijc11>3.0.co;2-d

[19] Livak KJ , Schmittgen TD . Analysis of relative gene expression data using real‐time quantitative PCR and the 2(‐Delta Delta C(T)) Method. Methods. 2001;25:402‐408. 11846609 10.1006/meth.2001.1262

[20] Lusky M . Good manufacturing practice production of adenoviral vectors for clinical trials. Hum Gene Ther. 2005;16:281‐291. 15812223 10.1089/hum.2005.16.281

[21] Shiseki M , Nagashima M , Pedeux RM , et al. p29ING4 and p28ING5 bind to p53 and p300, and enhance p53 activity. Cancer Res. 2003;63:2373‐2378. 12750254

[22] Ren X , Liu H , Zhang M , Wang M , Ma S . Co‐expression of ING4 and P53 enhances hypopharyngeal cancer chemosensitivity to cisplatin in vivo. Mol Med Rep. 2016;14(3):2431‐2438. 27484725 10.3892/mmr.2016.5552 PMC4991689

[23] Yang Y , Liu Y , He X , et al. ING4 alleviated lipopolysaccharide‐induced inflammation by regulating the NF‐kappaB pathway via a direct interaction with SIRT1. Immunol Cell Biol. 2020;98:127‐137. 31811786 10.1111/imcb.12308 PMC7384142

[24] Shao B , Liu E . Enzhong Liu. Expression of ING4 is negatively correlated with cellular proliferation and microvessel density in human glioma. Oncol Lett. 2017;14:3663‐3668. 28927128 10.3892/ol.2017.6618 PMC5587956

[25] Xie Y , Zhang H , Sheng W , Xiang J , Ye Z , Yang J . Adenovirus‐mediated ING4 expression suppresses lung carcinoma cell growth via induction of cell cycle alteration and apoptosis and inhibition of tumour invasion and angiogenesis. Cancer Lett. 2008;271:105‐116. 18789575 10.1016/j.canlet.2008.05.050

[26] Zhang X , Xu L‐S , Wang Z‐Q , et al. ING4 induces G2/M cell cycle arrest and enhances the chemosensitivity to DNA‐damage agents in HepG2 cells. FEBS Lett. 2004;570:7‐12. 15251430 10.1016/j.febslet.2004.06.010

[27] Li J , Martinka M , Li G . Role of ING4 in human melanoma cell migration, invasion and patient survival. Carcinogenesis. 2008;29:1373‐1379. 18375955 10.1093/carcin/bgn086

[28] Cai L , Liu J , Wang Y , et al. Enhanced anti‐melanoma efficacy of interferon alpha‐2b via overexpression of ING4 by enhanced Fas/FasL‐mediated apoptosis. Oncol Lett. 2018;15:9577‐9583. 29805679 10.3892/ol.2018.8534 PMC5958700

[29] Xie YF , Sheng W , Xiang J , Zhang H , Ye Z , Yang J . Adenovirus‐mediated ING4 expression suppresses pancreatic carcinoma cell growth via induction of cell‐cycle alteration, apoptosis, and inhibition of tumor angiogenesis. Cancer Biother Radiopharm. 2009;24:261‐269. 19409049 10.1089/cbr.2008.0582

[30] Unoki M , Shen JC , Zheng Z‐M , Harris CC . Novel splice variants of ING4 and their possible roles in the regulation of cell growth and motility. J Biol Chem. 2006;281:34677‐34686. 16973615 10.1074/jbc.M606296200

[31] Zhang X , Li J , Qin JJ , et al. Oncostatin M receptor beta deficiency attenuates atherogenesis by inhibiting JAK2/STAT3 signaling in macrophages. J Lipid Res. 2017;58:895‐906. 28258089 10.1194/jlr.M074112 PMC5408608

[32] Bruce AG , Hoggatt IH , Rose TM . Oncostatin M is a differentiation factor for myeloid leukemia cells. J Immunol. 1992;149:1271‐1275. 1380037

[33] Thoma B , Bird TA , Friend DJ , Gearing DP , Dower SK . Oncostatin M and leukemia inhibitory factor trigger overlapping and different signals through partially shared receptor complexes. J Biol Chem. 1994;269:6215‐6222. 8119965

[34] Junk DJ , Bryson BL , Smigiel JM , Parameswaran N , Bartel CA , Jackson MW . Oncostatin M promotes cancer cell plasticity through cooperative STAT3‐SMAD3 signaling. Oncogene. 2017;36:4001‐4013. 28288136 10.1038/onc.2017.33 PMC5509502

[35] Simonneau M , Frouin E , Huguier V , et al. Oncostatin M is overexpressed in skin squamous‐cell carcinoma and promotes tumor progression. Oncotarget. 2018;9(92):36457‐36473. 30559930 10.18632/oncotarget.26355 PMC6284862

[36] Shrivastava R , Asif M , Singh V , et al. M2 polarization of macrophages by Oncostatin M in hypoxic tumor microenvironment is mediated by mTORC2 and promotes tumor growth and metastasis. Cytokine. 2019;118:130‐143. 29625858 10.1016/j.cyto.2018.03.032

[37] Brounais B , Chipoy C , Mori K , et al. Oncostatin M induces bone loss and sensitizes rat osteosarcoma to the anti‐tumor effect of Midostaurin in vivo. Clin Cancer Res. 2008;14:5400‐5409. 18765531 10.1158/1078-0432.CCR-07-4781

[38] Bellido T , O'Brien CA , Roberson PK , Manolagas SC . Transcriptional activation of the p21(WAF1, CIP1, SDI1) gene by interleukin‐6 type cytokines. A prerequisite for their pro‐differentiating and anti‐apoptotic effects on human osteoblastic cells. J Biol Chem. 1998;273:21137‐21144. 9694869 10.1074/jbc.273.33.21137

[39] Wenbiao Z , Guan J . Oncostatin M promotes the osteogenic differentiation of mouse MC3T3‐E1osteoblasts through the regulation of monocyte chemotactic protein‐1. Mol Med Rep. 2018;18:2523‐2530. 30015860 10.3892/mmr.2018.9261 PMC6102744

[40] Ko HS , Park BJ , Choi SK , et al. STAT3 and ERK signaling pathways are implicated in the invasion activity by oncostatin M through induction of matrix metalloproteinases 2 and 9. Yonsei Med J. 2016;57:761‐768. 26996579 10.3349/ymj.2016.57.3.761 PMC4800369

[41] Damiens C , Grimaud E , Rousselle AV , et al. Cysteine protease production by human osteosarcoma cells (MG63, SAOS2) and its modulation by soluble factors. Cytokine. 2000;12:539‐542. 10857775 10.1006/cyto.1999.0593

[42] David E , Guihard P , Brounais B , et al. Direct anti‐cancer effect of oncostatin M on chondrosarcoma. Int J Cancer. 2011;128:1822‐1835. 21344373 10.1002/ijc.25776

[43] Han Z , Zhou C , Sun B , Yan Q , Zhang J . Experimental studies on the inhibition of adenovirus‐ING4‐OSM therapy on nasopharyngeal carcinoma proliferation in vitro and in vivo. Cell Biochem Biophys. 2014;70:1573‐1578. 25005773 10.1007/s12013-014-0097-z

